# You don’t know a person(’s taste) when you only know which genre they like: taste differences within five popular music genres based on sub-genres and sub-styles

**DOI:** 10.3389/fpsyg.2023.1062146

**Published:** 2023-06-07

**Authors:** Anne Siebrasse, Melanie Wald-Fuhrmann

**Affiliations:** Music Department, Max Planck Institute for Empirical Aesthetics, Frankfurt am Main, Germany

**Keywords:** musical taste, music genre, individual differences, personality traits, Sinus-Milieus

## Abstract

A representative German sample (*N* = 2,086) was surveyed on their musical taste with a questionnaire that asked about their liking not only of a number of genres, but also of relevant sub-genres and -styles. Using Latent Profile Analysis to analyze sub-genre liking patterns, four to six distinct taste classes were found within groups of those *n* = 1,749 people who liked either European classical music, electronic dance music, metal, pop or rock based on their sub-genre ratings. Across genres, two types of taste classes emerged: one with three classes that differed in the degree of liking all sub-genres, another with one to three classes that were biased in their liking or disliking of easier and more mainstream variants of a genre as compared to harder and sophisticated ones. Logistic regression models revealed meaningful relationships of genre fan groups and within-genre taste classes with sociodemographic variables and BIG-5 personality traits. In sum, our results demonstrate meaningful taste differences within genres and show that these translate to differences in person-related variables as well. These findings challenge earlier genre-based conceptualizations of music tastes, since we find similar structures already on the sub-genres level. It also suggests that different reasons and factors underlie tastes for genres and sub-genres. Future studies should therefore ask about taste in a more nuanced way.

## 1. Introduction

Tastes are known to be an important aspect of people’s identities and social lives. They can be seen as general, long-term attitudes that inform judgments and influence behavior. Originally, taste was a subject of philosophical discourse (Hume, 1757; Kant, 1790; Korsmeyer, 2013; Kivy, 2015). Accordingly, and in addition to the philosophical discourse (Hume, 1757; Kant, 1790; Korsmeyer, 2013; Kivy, 2015), the empirical study of tastes in artefacts and artworks, with regard to their content, structure, and effects, but also their development and influencing factors, has long been an important strand of psychological (Berlyne, 1974) and sociological (Farnsworth, 1950; Bourdieu, 1979; Schulze, 1992–2000; Gronow, 2001) research alike. Nevertheless, how best to measure and describe tastes in a reliable, nuanced, and meaningful way remains disputed.

In this paper, we focus on the case of taste in music. We demonstrate that the typical, very general approach to measuring such taste via lists of music genres comes with the drawback of overlooking meaningful differences. Specifically, we explore five taste groups: people who report liking European classical music, electronic dance music (EDM), metal, pop, and rock music, respectively. Most musical taste questionnaires do not allow further information regarding the content of people’s tastes to be obtained, assuming that similar genre ratings mean similar tastes. Here, however, we used a self-developed, musicologically informed questionnaire that also asked participants to indicate their knowledge of and liking for a number of related sub-genres and -styles. This allowed us to see that people who show the same rating behavior on the genre level may differ in their rating behavior on the level of sub-genres and -styles. We were also able to compare the degree to which taste differences across and within genres are related to personality traits and factors of social identity.

### 1.1. Measuring musical taste

On a conceptual level, musical taste is primarily understood as the types of music that people like and dislike, i.e., content. Categorizations of musical content mainly draw on categories that are invented and used by the music industry, musicians, critics and fans; they are used to sort music into what are commonly referred to as genres or styles, with super- and sub-genres and -styles also being acknowledged (Lena, 2012; Brackett, 2016).

There are several general approaches for quantitatively measuring actual musical tastes, but also taste-related preference behavior (Greasley and Lamont, 2016). Most often, researchers use questionnaires with lists of music category terms. The Musical Preference Scale (Litle and Zuckerman, 1986) or the Short Test of Musical Preferences (STOMP) and its revised version (STOMP-R) (Rentfrow and Gosling, 2003; Rentfrow et al., 2011) represent attempts to create standardized item batteries. Some studies have also used music clips meant to represent genres in addition to or instead of genre names, such as the Music Genres-Clips-Test (Bonneville-Roussy et al., 2017) or the excerpts used to establish the MUSIC model (Rentfrow et al., 2011, 2012). Only very few studies conceptualize musical taste on some other basis than that of genre, such as artist names (the Artist-based Musical Preferences measure; Ferrer et al., 2012) or genre-independent properties like melodic, rhythmic, harmonic and timbral characteristics or emotional expression (Schwartz and Fouts, 2003).

The advantages and disadvantages of each of these approaches have been discussed in the literature. In particular, genre-based measurement tools have been criticized because of the elusive and dynamic nature of genre terms, numbers, characteristics, and cultural associations, as well as the difficulties in distinguishing genres from super- and sub-genres (Greasley and Lamont, 2016; Brisson and Bianchi, 2020; Eggert, 2022). Although genre-based, the STOMP(−R) (Rentfrow and Gosling, 2003; Rentfrow et al., 2011) was meant to provide a general measurement tool and to overcome style- and genre-based descriptions of musical tastes by identifying higher-order factors with the help of dimension-reduction techniques. Its choice of musical categories, however, has been criticized from a musicological perspective inasmuch as they mix genres, sub-genres, styles, forms, and function-oriented categories (e.g., classical music and opera, with the latter being a form within the genre of classical music; religious music and soundtrack being defined not stylistically, but because of their function) and are biased towards Anglo-American music (Fleischer, 2012; see also Bonneville-Roussy et al., 2013; Brisson and Bianchi, 2020; Eggert, 2022). Accordingly, studies using the STOMP(−R) for non-Anglo-American samples omitted, added, or modified items to make it fit (Fricke and Herzberg, 2017; Warrener et al., 2020). Such modifications often led to factor solutions that differed in number and structure from the original factor structure, thus calling its validity into question (Chung et al., 2019; Brisson and Bianchi, 2020). However, even if no modifications are made and the original items of the STOMP are used, the original factor structure can possibly not be replicated, as in the study of Dunn et al. (2012). Lastly, broad genres—but even more so, the factors comprising several of them—reduce existing musical diversity to such an extent that it may no longer be possible to capture people’s actual tastes in a meaningful way (Greasley and Lamont, 2016).

### 1.2. Why sub-genres and sub-styles matter

Qualitative studies that allow people to characterize their musical taste in terms of how they themselves understand it show that people indeed refer to genres; but they also tend to go into more detail. They single out particularly appealing components of their generally liked genre(s) by naming musicians, composers, or performers, and by referring to musical elements, but also to sub-genre categories (Kunz, 1998; Greasley et al., 2013; Berli, 2014; Greb et al., 2017; Ackermann, 2019). The history of music is also full of cases of people drawing a very sharp line between types of music that might seem rather similar to outsiders: there is the notorious 19th-century “War of the Romantics” between classicist “Brahmins” and progressive Wagnerians (Walker, 1993, pp. 338–367); and there are opera fans who would never attend a string quartet concert and vice versa—nevertheless, all of these types of music fall into the category of classical music. Similarly, there are mutually exclusive fan groups for The Beatles and The Rolling Stones, both of which count as Rock‘n’Roll; and there are delineations in the world of metal that are hard to grasp by non-metalheads, but are matters of life and death for participants in their associated scenes (Walser, 1993; Chaker, 2014; Smialek, 2016).

Given the musical and societal, but also economic relevance of such within-genre demarcations, it seems worthwhile to consider them as well in empirical taste research (Vlegels and Lievens, 2017). Sub-genres and -styles can provide one possible starting point for this: they inhabit a middle ground between the too-granular level of individual artists or works and a genre as a whole. In addition, they reflect how insiders construct differences within a genre and come with related associations and meanings.

Sub-genres and -styles are features of many genres, first and foremost those with a longer history and with a particularly large number of exemplars. Sub-genres can evolve in succession or simultaneously. They can differ from each other in terms of one or more musical features, the instruments used, and the forms they take (such as masses and motets in Renaissance music vs. symphonies, solo concertos, and operas in the Romantic period of European classical music), as well as the style, content, and topics of any lyrics and libretti they may have, or performance aspects, such as playing and singing techniques. But they may also differ with regard to their underlying aesthetics and value systems and the social groups they appeal to. One could even say that sub-genres—just like genres—often create their own social milieu (Born, 2011).

### 1.3. Our study

Despite the large amount of historical and anecdotal evidence for the potential relevance of sub-genres for people’s musical taste, they are mostly absent in empirical taste research. So far, only very few studies include some sub-genres and -styles in their item lists, but in an unsystematic way and without explicitly acknowledging their nature as sub-genres (Otte, 2008; Rentfrow et al., 2011). To redress this omission, the present study explores whether people who like one of five large Western music genres (European classical music, electronic dance music, metal, pop, and rock) can be further distinguished into sub-groups on the basis of their attitudes towards sub-genres of the respective genres. The sub-groups were identified using Latent Profile Analysis (LPA), a person-centered approach to uncovering latent groups that show a similar rating behavior on a number of ordinal or continuous variables.

Tastes, like music genres and sub-genres as well, have an inherently social component (Schulze, 1992–2000; Brackett, 2016). At the same time, people see their own and others’ tastes as expressions of personality and individuality, thus constituting taste publics (Fox and Wince, 1975; Hargreaves, 1986, p. 179ss.; Shepherd, 2003). Accordingly, there are sociological and psychological research strands that attempt to correlate tastes with sociodemographic and personality variables. We therefore analyzed whether the taste differences of sub-groups were also related to sociodemographic and psychological differences, including Sinus-Milieus, a lifestyle measure that had never before been used in research on musical taste (SINUS Markt- und Sozialforschung GmbH, 2018). The data for this study came from a representative survey on musical taste in Germany with *N* = 2,086 participants.

## 2. Materials and methods

### 2.1. Participants and procedure

This study contains data from a subsample of *n* = 1,749 participants (49.0% female, 51.0% male, *M*_age_ = 48.0 years, *SD*_age_ = 17.4, age range: 18–94) of a total of *N* = 2,086 participants, who were surveyed on various aspects of their musical taste via a mixed-methods procedure using online or computer-assisted personal interviews (CAPI). This approach was chosen to survey a sample that was representative in terms of age, gender, education, and state of residence for the German population aged 18 and above. We commissioned the Ipsos Institute to collect the data from their own panel. Data collection took place from December 2016 to February 2017. Participants in the online study (*n* = 1,204) were invited via e-mail after being selected by the sampling tool Samplix, which randomly draws a sample from a panel of 160,000 people using the above quotas mentioned above. For CAPI (*n* = 882), trained and supervised interviewers interviewed the target persons via telephone. The average duration of the survey was 20.0 min (online) and 38.5 min (CAPI). Participants received no fee or recompensation.

### 2.2. Measures

#### 2.2.1. Musical taste

Musical taste was measured as the degree to which participants reported to know, like, or dislike music genres and sub-genres. For this, we created our own inventory, not only because the surveyed population was German, but mainly because of musicological inconsistencies in existing, supposedly genre-based questionnaires of musical taste. To create an exhaustive genre-based musical taste questionnaire that meets musicological standards and mirrors present categorization practices and customs in Germany, musical genres and their sub-genres were compiled from musicological encyclopedias, as well as music magazines, webpages, and record shops in several large German cities. Those genres and sub-genres that appeared in the majority of sources and could be described on the basis of stylistic features were included on the questionnaire. The musical taste questionnaire as a whole was subjected to a pre-test (*N* = 3,318, *M*_age_ = 33.5 years, *SD*_age_ = 25.5, 40% female; online survey, convenience sample). Participants rated their familiarity and liking of 18 genres and 8–19 related sub-genres and had the opportunity to add genres and sub-genres that they felt were missing. Based on this data, we excluded those genres and terms from our final questionnaire that were not known to more than 15% of participants (i.e., Latin, New Age, Reggae, World Music, and Traditional music of other cultures; the latter two being merged into Non-European Music in the final questionnaire, although it is clear that this is not a style-based category) and of those sub-genres that were most frequently not known per genre. The final questionnaire consisted of 14 genres, with 11 of them having 8–19 sub-genres.

Participants rated genres and sub-genres by either saying they did not know it or indicating the degree to which they liked it using a five-point Likert scale ranging from “do not like at all” (1) to “neutral” (3) to “like particularly well” (5). Participants were asked to rate sub-genres only for the genres they knew.

Other music-related attitudes (e.g., interest in searching for unfamiliar music, functions of music use) and behaviors (e.g., sources used to search for unfamiliar music, situations for engaging with music) were assessed, but not used to address the present research.

#### 2.2.2. Listening frequency

Listening frequency was assessed for all 14 genres with the item “Please indicate how often you actually listen to the following musical styles (this refers only to situations in which you can choose yourself which music is played).” Response scale was a five-point Likert scale ranging from 1 “never” to 5 “daily.”

#### 2.2.3. Sociodemographics

The questionnaire contained detailed information on sociodemographic variables, of which we used age, gender (male, female, diverse), education level and Sinus-Milieu to address the research question of this article. Sinus-Milieus are models of social groups developed by the SINUS-Institut, a market and social research company based in Berlin and Heidelberg, to which people are assigned on the basis of their lifestyle and attitudes (Barth et al., 2018). Ten different milieus are mapped onto the two dimensions of socioeconomic status (SES) and basic attitudes, i.e., SES low: Precarious (PRE), Hedonists (HED), Traditionals (TRA); SES middle: Modern Mainstreamers (MMS), Adaptive Navigators (ADA), Social Ecologicals (SOC); SES high: Established (EST), Liberal Intellectuals (LIB), Performers (PER), Cosmopolitan Avant-gardes (COS); attitudes tradition: TRA, EST, PRE, MMS; modernization: SOC, LIB; and reorientation: PER, COS, ADA, HED (SINUS Markt- und Sozialforschung GmbH, 2018). The distribution of the Sinus Milieus for the entire sample and the subgroups can be found in Supplementary Figures S1–S6.

#### 2.2.4. Personality traits

To assess participants’ personality traits, the German version of the 10-item Big Five Inventory was used (Rammstedt and John, 2007). Here, the dimensions neuroticism, extraversion, openness to experience, agreeableness and conscientiousness are measured with two items each.

### 2.3. Statistical analyses

#### 2.3.1. Data pre-processing and preparation

In order to identify possible sub-style based groups within “fans” of certain genres, we filtered for those participants who liked at least one of five genres of interest (European classical music, electronic dance music, metal, pop, and rock). To decide on the genres to be tested, we first excluded the two genres that are relevant only or primarily in Germany (Schlager, German folk music). In addition, we excluded the genres for which we could not identify a (sufficient) number of sub-styles (rap and funk). Of the remaining, we selected those genres that were most liked and most widely known (pop, rock, and classical). In order to include also less popular genres in the analysis we selected two genres that had very low familiarity and liking values (EDM and metal) but are known from the literature to have relevant discourses and practices of sub-style differentiation. For more information, see Supplementary Table S1. Subsamples were generated by coding for each participant, regarding whether they had given a liking rating of 4 or 5 to each of the five genres of interest, which resulted in a subsample of *n* = 1,749.

The groups of participants who liked a genre were then subjected to latent profile analyses (LPAs). For the LPAs within these groups the answers “I do not know the term” and “I do not know” were combined as “sub-style unknown” and defined as missing values.”

Participants who selected “sub-style unknown” for all sub-styles were excluded from the LPAs. The final samples counted classic *n* = 701, EDM *n =* 469, metal *n* = 389, pop *n =* 1,345, and rock *n =* 1,167. Of those, *n* = 414 liked only one of the genres, *n* = 607 liked two genres, *n* = 472 liked three genres, and *n* = 259 liked four to five genres (see Supplementary Figure S7). Supplementary Table S2 shows mean values and standard deviations for genre preference, listening frequency, and sub-genre knowledge.

#### 2.3.2. Latent profile analyses

LPA as a probabilistic person-centered approach focuses on patterns of attitudes and thus can be used to identify latent sub-groups (or “classes”) of musical taste based on similar liking ratings on the sub-style level. We inspected a series of LPA models with one through six classes for the taste groups of EDM, metal, pop, and rock music and one through seven classes for the classic music group. To begin with, all models were estimated using 500 starting values and 50 iterations, and were adjusted up to 1,000 starting values and 100 iterations when the best Log Likelihood value was not replicated. To determine the best number of profiles, we considered the following statistical fit indices: Bayesian Information Criterion (BIC), Sample-Size Adjusted BIC (SABIC), and Akaike’s Information Criterion (AIC), where lower values indicate better fit (Nylund et al., 2007); and entropy as a measure of classification accuracy, which can range from 0 to 1, with higher values representing a better fit of the model. To compare each model to a model with one less profile, we conducted likelihood-based tests, both the Lo–Mendell–Rubin (LMR) test and the bootstrap likelihood ratio test (BLRT). The *value of p* of a likelihood ratio test (LRT) gives the likelihood that the data have been generated by a model with one class less. Masyn (2013) mentions the problem that LRTs may never become significant and suggests looking at the relative decreases of the information criteria in such a case. In addition, we examined the estimated class sizes, as it is recommended to reject model solutions with classes that contain less than 5% of the sample (Ferguson et al., 2020). Also, to allow for reliable inference statistical analyses, we wanted classes to have at least 30 members.

We emphasized theoretical considerations in terms of interpretability of the profiles and especially the identification of meaningful groupings. Therefore, we investigated the shape of profiles to identify the models with distinct and meaningful patterns. M*plus* version 8.4 (Muthén and Muthén, 1998–2017) was used to estimate all models.

#### 2.3.3. Logistic regression analyses

In order to investigate relations between musical taste and sociodemographic and personality variables, we conducted logistic regression analyses on the genre level and within the genres on the level of classes. On the genre level, we used binomial logistic regressions with liking the genre (=1) vs. not liking the genre (=0). On the class level, polynomial logistic regressions were computed. It was not possible to use polynomial logistic regression analyses for genre comparison as well, given that the majority of participants belonged to more than one genre group. Regression analyses were conducted using IBM SPSS statistics (versions 26 and 28).

## 3. Results

### 3.1. Latent profile analyses

**Figure 1:**
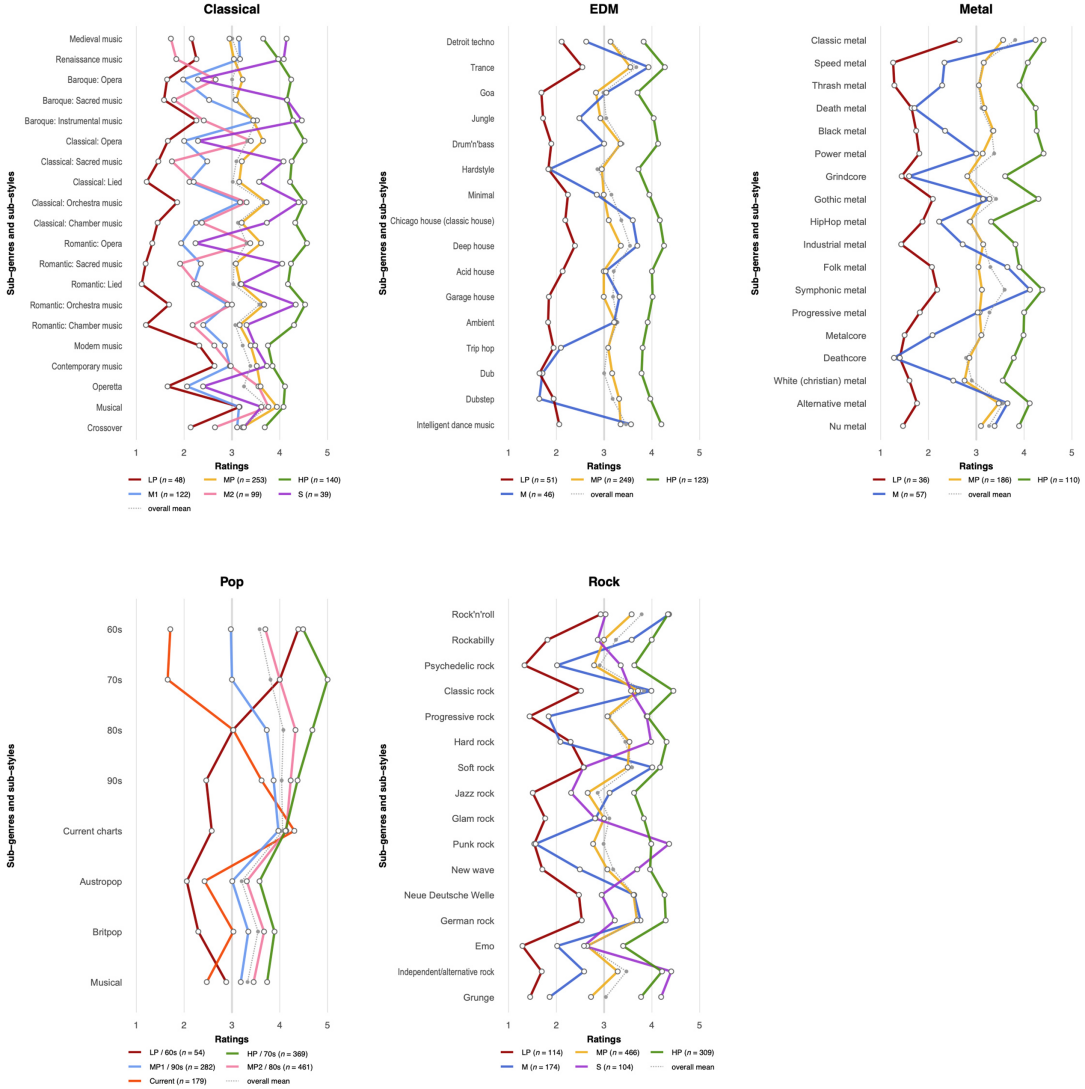
Liking profiles for taste classes of five music genres. LP, low preference class; MP, medium preference class; HP, high preference class; M, mainstream/soft class; S, sophisticated/hard class.

Mean liking ratings for sub-genres range between 3.00 and 3.74 (classical), 3.00 and 3.70 (EDM), 2.79 and 3.81 (metal), 3.20 and 4.08 (pop), and 2.66 and 3.84 (rock) (see Figure 1). Similar patterns emerged across genres: sub-genres and -styles that form something like the core, standard, or prototype of the respective genre are most liked, such as baroque, classical, and romantic orchestra music, classic metal, pop from the 80s to the current charts, Rock and roll and classic rock. Also, more “digestible,” easier-to-process sub-genres, such as musicals, trance, deep house, symphonic metal, soft rock, or Neue deutsche Welle, are generally preferred over more challenging variants.

#### 3.1.1. Model retention decisions

The fit statistics for the LPAs are displayed in Table 1. Values that indicate better model fit are given in boldface. Concerning the pop group, the decision was quite clear, since fit indices and theoretical considerations went hand in hand: Model 5 was retained as the best model to fit the data based on the low absolute Log likelihood value; AIC, BIC, and SABIC values; and both significant LMR and BLRT tests. Entropy is above 0.90 for Model 4, Model 5, and Model 6 and nearly the same in these models. Also, the profiles of the model are distinct and well interpretable. For classical, metal, and rock, our findings are ambiguous regarding the optimal model: AIC, BIC and SABIC are decreasing; the LMR test prefers no model for metal; Model 3 for classical and Model 4 for rock and entropy values differ. However, the mean profiles of the respective models reveal distinct and meaningful classes that make sense from a theoretical perspective. Hence, we retained Model 6 for classical music, Model 4 for metal, and Model 5 for rock. In the EDM group, fit indices point to a three-profile solution, but Model 4 provides a more differentiating profile for the additional class. As the new class was sufficiently large, we retained Model 4 for further investigations.

**Table 1:** Model comparisons for latent profile analyses.

Genre	Model	Log likelihood	AIC	BIC	SABIC	Entropy	LMR *p*-value	BLRT *p*-value	*n* per class
Classical	1	−18140.770	36,361.540	36,543.640	36,416.632				*n* = 701
	2	−16507.432	33,136.865	33,414.568	33,220.880	**0.87**	<0.001	<0.001	*n*_1_ = 372; *n*_2_ = 329
	3	−15969.469	32,102.938	32,476.244	32,215.877	0.85	**<0.001**	<0.001	*n*_1_ = 169; *n*_2_ = 322; *n*_3_ = 210
	4	−15784.102	31,774.203	32,243.111	31,916.065	**0.87**	0.151	<0.001	*n*_1_ = 157; *n*_2_ = 279; *n*_3_ = 64; *n*_4_ = 201
	5	−15606.193	31,460.385	32,024.896	31,631.171	0.85	0.736	<0.001	*n*_1_ = 110; *n*_2_ = 115; *n*_3_ = 250; *n*_4_ = 72; *n*_5_ = 154
	**6**	−15452.087	31,194.174	31,854.287	31,393.883	**0.87**	0.227	<0.001	*n*_1_ = 48; *n*_2_ = 122; *n*_3_ = 99; *n*_4_ = 253; *n*_5_ = 140; *n*_6_ = 39
	7	−15329.275	**30,990.550**	**31,746.267**	**31,219.183**	0.86	0.195	<0.001	*n*_1_ = 45; *n*_2_ = 79; *n*_3_ = 217; *n*_4_ = 146; *n*_5_ = 123; *n*_6_ = 38; *n*_7_ = 53
EDM	1	−6904.229	13,872.457	14,005.276	13,903.715				*n* = 469
	2	−6490.135	13,078.269	13,281.649	13,126.132	0.66	0.223	<0.001	*n*_1_ = 204; *n*_2_ = 265
	3	−6318.022	12,768.045	13,041.984	12,832.514	**0.75**	**0.001**	<0.001	*n*_1_ = 289; *n*_2_ = 57; *n*_3_ = 123
	**4**	−6262.223	12,690.446	**13,034.946**	12,771.520	0.72	0.711	<0.001	*n*_1_ = 249; *n*_2_ = 46; *n*_3_ = 51; *n*_4_ = 123
	5	−6212.774	**12,625.547**	13,040.608	**12,723.228**	0.73	0.135	<0.001	*n*_1_ = 48; *n*_2_ = 207; *n*_3_ = 53; *n*_4_ = 149; *n*_5_ = 12
Metal	1	−7905.317	15,882.634	16,025.323	15,911.097				*n* = 389
	2	−7386.044	14,882.089	15,100.086	14,925.575	0.78	0.128	<0.001	*n*_1_ = 162; *n*_2_ = 227
	3	−7147.723	14,443.446	14,736.751	14,501.955	**0.83**	0.120	<0.001	*n*_1_ = 74; *n*_2_ = 220; *n*_3_ = 95
	**4**	−7054.540	14,295.079	14,663.692	14,368.610	0.82	0.564	<0.001	*n*_1_ = 36; *n*_2_ = 186; *n*_3_ = 57; *n*_4_ = 110
	5	−6960.155	14,144.310	**14,588.231**	14,232.863	0.80	0.468	<0.001	*n*_1_ = 54; *n*_2_ = 33; *n*_3_ = 141; *n*_4_ = 131; *n*_5_ = 30
Pop	1	−13618.875	27,269.751	27,353.017	27,302.192				*n* = 1,345
	2	−12894.161	25,838.323	25,968.427	25,889.012	0.81	<0.001	<0.001	*n*_1_ = 363; *n*_2_ = 982
	3	−12620.150	25,308.299	25,485.241	25,377.237	0.81	0.007	<0.001	*n*_1_ = 288; *n*_2_ = 851; *n*_3_ = 206
	4	−11986.639	24,059.279	24,283.057	24,146.465	**0.94**	0.002	<0.001	*n*_1_ = 180; *n*_2_ = 369; *n*_3_ = 522; *n*_4_ = 274
	**5**	−11858.903	23,821.806	24,092.422	23,927.241	0.93	**0.003**	<0.001	*n*_1_ = 54; *n*_2_ = 179; *n*_3_ = 282; *n*_4_ = 461; *n*_5_ = 369
	6	−11510.361	**23,142.721**	**23,460.174**	**23,266.404**	0.92	0.100	<0.001	*n*_1_ = 96; *n*_2_ = 99; *n*_3_ = 59; *n*_4_ = 197; *n*_5_ = 411; *n*_6_ = 483
Rock	1	−21892.432	43,848.864	44,010.854	43,909.211				*n* = 1,167
	2	−20802.864	41,703.728	41,951.775	41,796.134	0.75	0.002	<0.001	*n*_1_ = 433; *n*_2_ = 734
	3	−20416.717	40,965.434	41,299.539	41,089.900	**0.77**	0.007	<0.001	*n*_1_ = 130; *n*_2_ = 616; *n*_3_ = 421
	4	−20175.987	40,517.974	40,938.136	40,674.499	0.74	**0.009**	<0.001	*n*_1_ = 126; *n*_2_ = 343; *n*_3_ = 355; *n*_4_ = 343
	**5**	−20067.625	40,335.251	40,841.470	40,523.836	0.74	0.608	<0.001	*n*_1_ = 114; *n*_2_ = 174; *n*_3_ = 466; *n*_4_ = 104; *n*_5_ = 309
	6	−19958.425	**40,150.850**	**40,743.127**	**40,371.495**	0.72	0.330	<0.001	*n*_1_ = 104; *n*_2_ = 229; *n*_3_ = 260; *n*_4_ = 340; *n*_5_ = 105; *n*_6_ = 129

AIC, Akaike’s Information Criterion; BIC, Bayesian Information Criterion; SABIC, sample-size adjusted BIC; LMP, Lo–Mendell–Rubin test; BLRT, bootstrap likelihood ratio test. The smallest values for information criteria and *p*-values of LRTs and the largest values for entropy, as well as the retained model are boldfaced.

#### 3.1.2. Sub-genre taste profiles

Across genres, we found comparable patterns: the sub-genre preference profile of the largest class per genre is very similar to the overall mean curve, while two further classes mirror this curve on lower and higher levels—the class with the lowest means typically being the (second) smallest, and the one with the highest means overall being the second largest. We called these classes the *low*, *medium*, and *high preference classes*.

The remaining classes, however, show a differentiated pattern with the poles mainstream/soft/easier-to-process vs. sophisticated/hard/intellectually and perceptually challenging (see Figure 1). The three differentiated taste profiles within the classical music group differ mainly with regard to their liking or disliking of opera and sacred music. One class is overall very similar to the high preference group, but shows a clear dislike for opera and operetta. Another class shares this dislike, but also dislikes sacred and chamber music, while reporting only medium preferences for orchestral music, early music, and new music, as well as musicals. A third differentiated class, on the contrary, likes nothing but musicals, operetta, and opera along with Classical and Romantic orchestral music, albeit only to a medium degree. Accordingly, the two latter classes manifest as different expressions of the mainstream/soft/easier pole of the second type, whereas the remaining class appears to be positioned at the sophisticated end.

The differentiated class of the EDM group likes only some of the sub-genres most preferred by the medium and high classes (i.e., some trance and house sub-styles that are currently quite popular and emphasize a regular 4/4 beat), while showing a strong dislike of downbeat and hardcore techno sub-styles (i.e., dub, dubstep, trip hop, and hardstyle, variants with broken-beat rhythms and/or minimal arrangements); it could therefore be interpreted as belonging to the mainstream/soft/easier pole. In metal, the differentiated class also likes the more mainstream and softer sub-styles, such as classic, folk, and symphonic metal, while rejecting harder and extreme sub-styles, such as black and death metal, deathcore, and grindcore. A class that prefers the classic and more digestible sub-styles, while disliking their harder variants, is also evident for rock. Here, however, a contrasting taste class emerged that gives its highest ratings to the harder and more sophisticated sub-genres (i.e., hard rock, punk rock, grunge, alternative, and progressive rock), while disliking the softer ones.

A different picture emerged with the pop music group: here, all but one class (low preference/1960s) converge at their preference for the current charts, but differ strongly with regard to how much they (dis)like older forms, with either the 1970s, 1980s, 1990s, or current charts being their most preferred period of pop music.

Supplementary Figure S8 shows how many of those participants who like two or three of the five genres belong to the same class or type across genres.

### 3.2. Relationships with sociodemographic and personality variables

In the following, we report results of associations between taste groups and sociodemographic and personality variables, first on the level of genre groups, and then across sub-genre taste classes on the basis of logistic regression models. Descriptive statistics can be found in Table 2 (genre level) and Table 3 (class level); regression models can be found in Table 4 (genre level) and Table 5 (class level).

**Table 2:** Sociodemographic and personality values for genre groups.

	Sociodemographic variables					Personality traits				
Group	Age (*M*)	Gender (% female)	Education (*M*)	Sinus Milieu, SES: low, middle, and high (%)	Sinus Milieu, attitudes: tradition, modernization, and reorientation (%)	Extraversion (*M*)	Neuroticism (*M*)	Openness (*M*)	Conscientiousness (*M*)	Agreeableness (*M*)
Classical (*n* = 701)	53.6	52.4	2.2	31.4/27.9/40.7	43.4/20.1/36.4	3.2	2.6	3.6	3.8	3.2
Non-liking (*n* = 1,048)	44.3	46.8	2.0	35.0/30.9/34.1	39.4/12.8/47.8	3.2	2.7	3.2	3.7	3.2
EDM (*n* = 469)	37.2	46.1	2.3	30.9/29.2/39.9	27.5/13.2/59.3	3.3	2.7	3.4	3.6	3.1
Non-liking (*n* = 1,280)	52.0	50.1	2.0	34.6/29.9/35.6	46.0/16.7/37.3	3.1	2.7	3.3	3.8	3.2
Metal (*n* = 389)	41.3	39.3	2.3	35.0/25.7/39.3	25.7/15.9/58.4	3.1	2.7	3.6	3.6	3.1
Non-liking (*n* = 1,360)	50.0	51.8	2.0	33.2/30.8/36.0	45.4/15.7/38.9	3.2	2.6	3.3	3.8	3.2
Pop (*n* = 1,345)	46.1	51.4	2.1	30.7/30.8/38.5	38.3/16.0/45.7	3.2	2.7	3.4	3.7	3.2
Non-liking (*n* = 404)	54.5	41.1	2.0	43.3/26.0/30.7	50.0/14.9/35.1	3.1	2.6	3.3	3.7	3.2
Rock (*n* = 1,167)	46.5	45.8	2.2	30.6/29.9/39.5	35.8/17.7/46.6	3.2	2.7	3.4	3.7	3.2
Non-liking (*n* = 582)	51.1	55.5	2.0	39.5/29.2/31.3	51.5/11.9/36.6	3.2	2.7	3.2	3.7	3.3

Values for groups not liking the genre are given for comparison purposes for the binary regression analyses.

**Table 3:** Sociodemographic and psychological values for taste classes.

	Sociodemographic variables					Personality traits				
Class	Age (M)	Gender (% female)	Education	Sinus milieu, SES: low, middle, and high (%)	Sinus milieu, attitudes: tradition, modernization, and reorientation (%)	Extraversion	Neuroticism	Openness	Conscientiousness	Agreeableness
Classical										
Low (*n* = 48)	52.6	39.6	2.0	43.8/27.1/27.1	56.3/16.7/25	3.3	2.6	3.3	3.9	3.1
Mainstream 1 (*n* = 122)	51.7	48.4	2.1	35.2/32.8/32.0	44.3/20.5/35.2	3.1	2.7	3.4	3.6	3.3
Mainstream 2 (*n* = 99)	59.7	52.5	2.0	33.3/22.2/44.4	51.5/19.2/29.3	3.4	2.5	3.5	4.0	3.2
Medium (*n* = 253)	53.5	56.1	2.2	29.6/25.7/44.7	43.5/19.4/37.2	3.1	2.7	3.6	3.8	3.2
High (*n* = 140)	53.2	56.4	2.4	25.0/33.6/41.4	34.3/22.1/43.6	3.3	2.7	3.8	3.8	3.3
Sophisticated (*n* = 39)	47.9	41.0	2.2	33.3/20.5/46.2	35.9/23.1/41.0	2.9	2.3	4.0	3.9	3.3
Δ	11.8	16.8	0.4	19.7/13.1/18.5	22.1/6.1/18.1	0.5	0.4	0.7	0.4	0.2
EDM										
Low (*n* = 51)	34.2	43.1	2.3	31.4/35.3/33.3	33.3/3.9/62.7	3.3	2.6	3.3	3.7	2.9
Medium (*n* = 249)	37.5	48.2	2.3	30.1/32.5/37.3	32.1/16.5/51.4	3.3	2.8	3.4	3.6	3.2
Mainstream/soft (*n* = 46)	33.9	50.0	2.1	37.0/17.4/45.7	19.6/6.5/73.9	3.5	2.6	3.3	3.3	3.1
High (*n* = 123)	39.2	41.5	2.3	30.1/24.4/45.5	18.7/13.0/68.3	3.3	2.5	3.6	3.7	3.1
Δ	5.3	8.5	0.2	6.9/17.9/12.4	15.6/9.1/22.5	0.2	0.3	0.3	0.4	0.3
Metal										
Low (*n* = 36)	42.6	38.9	2.0	44.4/36.1/19.4	19.4/27.8/52.8	3.1	2.7	3.5	3.9	3.0
Medium (*n* = 186)	42.8	32.8	2.2	30.1/26.3/43.5	29.0/14.5/56.5	3.2	2.7	3.5	3.5	3.0
Mainstream/soft (*n* = 57)	39.5	52.6	2.5	35.1/15.8/49.1	35.1/14.0/50.9	3.0	2.6	3.7	3.4	3.3
High (*n* = 110)	39.1	33.3	2.4	40.0/26.4/33.6	17.3/15.5/67.3	3.1	2.8	3.7	3.6	3.1
Δ	3.7	9.6	0.5	14.3/20.3/29.7	15.7/13.8/16.4	0.2	0.2	0.2	0.5	0.3
Pop										
Low, peak at 60s (*n* = 54)	62.1	48.1	1.6	55.6/16.7/27.8	72.2/1.9/25.9	3.1	2.6	3.2	3.5	3.3
Peak at 80s (*n* = 461)	47.1	52.5	2.1	28.3/30.4/41.3	38.0/18.0/43.9	3.2	2.6	3.5	3.8	3.2
Medium, peak at 90s (*n* = 282)	40.8	52.8	2.2	27.0/33.3/39.7	37.6/14.5/47.9	3.1	2.7	3.3	3.6	3.1
Peak at charts (*n* = 179)	32.6	52	2.0	40.2/29.1/30.7	27.4/11.7/60.9	3.2	2.8	3.0	3.4	3.2
High, peak at 70s (*n* = 369)	53.1	49.1	2.1	28.2/32.2/39.6	39.6/18.7/41.7	3.2	2.6	3.5	3.8	3.2
Δ	29.5	3.2	0.6	28.6/16.6/13.5	44.8/16.8/35.0	0.1	0.2	0.5	0.4	0.2
Rock										
Low (*n* = 114)	45.3	39.5	2.0	33.3/30.7/36.0	46.5/14.9/38.6	3.2	2.4	3.3	3.8	3.2
Mainstream/soft (*n* = 174)	54.8	51.7	2.0	25.3/32.2/42.5	44.3/24.7/31.0	3.2	2.6	3.4	3.9	3.3
Medium (*n* = 466)	45.5	44.6	2.1	31.3/30.5/38.2	36.3/15.7/48.1	3.1	2.7	3.3	3.6	3.2
Sophisticated/hard (*n* = 104)	37.7	47.6	2.5	37.5/27.9/34.6	25.0/14.4/60.6	3.0	2.8	3.6	3.4	2.9
High (*n* = 309)	46.9	46.9	2.2	29.2/28.2/42.5	29.9/18.8/51.3	3.3	2.7	3.6	3.7	3.1
Δ	17.1	7.2	0.5	12.2/4.3/10.9	21.5/10.3/19.6	0.2	0.4	0.3	0.5	0.4

**Table 4:** Results of logistic regressions for genre groups.

	Classical			EDM			Metal			Pop			Rock		
Predictor	B (coef.)	OR	CI 95% (LL–UL)	B (coef.)	OR	CI 95% (LL–UL)	B (coef.)	OR	CI 95% (LL–UL)	B (coef.)	OR	CI 95% (LL–UL)	B (coef.)	OR	CI 95% (LL–UL)
*Sociodemographic variables*															
Age	0.049***	**1.050**	**1.042–1.058**	−0.053***	**0.949**	**0.941–0.957**	−0.026***	**0.975**	**0.967–0.983**	−0.027***	**0.973**	**0.965–0.981**	−0.011*	**0.989**	**0.983–0.996**
Gender: female	0.162	1.176	0.942–1.469	−0.279*	**0.756**	**0.591–0.968**	−0.682***	**0.506**	**0.391–0.653**	0.371*	**1.449**	**1.136–1.848**	−0.524***	**0.592**	**0.476–0.738**
Education	0.647***	**1.909**	**1.636–2.228**	0.134	1.144	0.973–1.344	0.095	1.100	0.932–1.298	−0.078	0.925	0.788–1.085	0.181*	**1.198**	**1.039–1.382**
Milieu, SES: medium	−0.185	0.831	0.625–1.105	0.187	1.205	0.875–1.659	−0.232	0.793	0.570–1.104	0.516***	**1.676**	**1.237–2.270**	0.144	1.155	0.880–1.514
Milieu, SES: high	0.030	1.030	0.779–1.363	0.052	1.053	0.775–1.433	−0.183	0.833	0.611–1.135	0.543***	**1.722**	**1.270–2.333**	0.188	1.207	0.918–1.587
Milieu, attitude: modernization	0.338*	**1.403**	**1.007–1.953**	−0.026	0.974	0.664–1.430	0.496*	**1.643**	**1.110–2.430**	−0.054	0.947	0.656–1.369	0.500*	**1.648**	**1.173–2.315**
Milieu, attitude: reorientation	−0.119	0.888	0.687–1.147	0.318*	**1.375**	**1.041–1.816**	0.610***	**1.841**	**1.374–2.466**	0.101	1.107	0.837–1.464	0.349*	**1.418**	**1.108–1.816**
*Personality traits*															
BFI-E	−0.024	0.976	0.863–1.103	0.232**	**1.262**	**1.104–1.816**	−0.057	0.944	0.825–1.081	0.135*	**1.144**	**1.002–1.306**	−0.028	0.972	0.862–1.096
BFI-N	0.105	1.111	0.971–1.271	−0.065	0.937	0.806–1.089	0.019	1.019	0.875–1.186	0.058	1.060	0.916–1.226	0.038	1.039	0.909–1.187
BFI-O	0.512***	**1.668**	**1.458–1.909**	0.055	1.057	0.914–1.222	0.422***	**1.524**	**1.311–1.773**	−0.060	0.942	0.817–1.085	0.308***	**1.361**	**1.196–1.549**
BFI-C	−0.068	0.934	0.809–1.078	−0.022	0.978	0.837–1.143	−0.178*	**0.837**	**0.714–0.981**	−0.022	0.978	0.838–1.141	0.024	1.024	0.892–1.176
BFI-A	0.161*	**1.175**	**1.016–1.358**	−0.127	0.881	0.750–1.035	−0.209*	**0.811**	**0.689–0.955**	−0.023	0.978	0.837–1.141	−0.144*	**0.866**	**0.751–0.998**
*χ*^2^ (df)	316.443(12)***			281.638(12)***			183.255(12)***			101.674(12)***			122.155(12)***		
Nagelkerke’s *R*^2^	0.228			0.223			0.155			0.087			0.096		
Correctly predicted cases in %	67.6			74.4			77.0			77.2			70.7		

Liking the genre (=1) versus not liking it (=0). CI, confidence interval; OR, odds ratio; LL, lower limit; UL, upper limit. ***p ≤ 0.0001; **p ≤ 0.001; *p ≤ 0.05. n = 1,709. Significant values are boldfaced.

**Table 5:** Results of polynomial regression models within genres.

	Classical (*n* = 690)		EDM (*n* = 446)		Metal (*n* = 382)		Pop (*n* = 1,308)		Rock (*n* = 1,145)	
	*p*	Classes that differ significantly from reference class, (OR)	*p*	Classes that differ significantly from reference class (OR)	*p*	Classes that differ significantly from reference class (OR)	*p*	Classes that differ significantly from reference class (OR)	*p*	Classes that differ significantly from reference class (OR)
*Sociodemographic variables*										
Age	**0.001**	**lp (0.962), m1 (0.970), s (0.970)**	0.140		0.379		**< 0.001**	**lp (1.028), mp1 (0.943), mp2 (0.971), current (0.907)**	**< 0.001**	**lp (0.981), m (1.032), s (0.964)**
Gender	0.139		0.758		0.137		0.835		0.126	
Education	**0.003**	**lp (0.561), m1 (0.497), m2 (0.589), mp (0.688), s (0.473)**	0.453		0.025		**0.011**	**current (0.637)**	0.043	
Milieu: SES	0.209		0.688		**< 0.001**	**mp (low: 0.375), m (middle: 0.284)**	0.104		0.890	
Milieu: attitude	0.457	lp (trad: 3.421**)	**0.014**	**mp (trad: 2.159)**	**0.026**	**m (trad: 2.928)**	0.166		**0.037**	**lp (trad: 2.203), m (trad: 1.692)**
*Personality traits*										
BFI-E	**0.031**	**s (0.576)**	0.534		0.638		0.351		0.379	lp (0.744*)
BFI-N	0.469	s (0.631*)	0.254		0.420		0.693		0.249	
BFI-O	**< 0.001**	**lp (0.504), m1 (0.602), m2 (0.621), mp (0.659)**	0.088	mp (0.709*)	0.082	*mp (0.696)*	**< 0.001**	**mp1 (0.794), current (0.524)**	**< 0.001**	**lp (0.627), m (0.654), mp (0.718)**
BFI-C	0.119		0.151		**0.003**	**lp (1.839), m (0.587)**	0.174	lp (0.636*)	0.058	
BFI-A	0.641		0.084		0.206		0.291		0.092	
χ^2^ (df)	132.178(60)***		62.024(36)*		87.136(36)***		384.017(48)***		199.136(48)***	
Nagelkerke’s *R*^2^	0.182		0.144		0.224		0.270		0.169	

Reference class: high preference class. OR, odds ratio. Abbreviations for classes: lp, low preference class; mp, medium preference class; m, mainstream/soft preference class; s, sophisticated/hard preference class. In italics: effects for predictors that are not significant in the overall model, but in a single class comparison. ***p ≤ 0.0001; **p ≤ 0.001; *p ≤ 0.05. Significant values are boldfaced.

Sociodemographic variables were: age, gender, education level (low, medium, and high), socioeconomic status (low, middle, and high), and milieu-related attitude (tradition, modernization, and reorientation). The two latter variables were derived from the Sinus Milieu classification of each participant. As a measure of personality, we used the BIG-5 personality traits.

#### 3.2.1. Comparison on genre level

When we compared people who like a genre with those who do not, we found three to six variables to be relevant predictors; these were most often: age, gender, milieu-related attitude, openness, and agreeableness (see Table 4). While the models for classical music, EDM, and metal have moderate Nagelkerke’s *R*^2^ values between 0.155 and 0.228, the pop and rock models have only weak explanatory power (*R*^2^ values <0.100; interpretation follows Cohen, 1988). People who like classical music are typically older, better educated, more open and agreeable and more often have a milieu-related attitude oriented towards modernization than people who do not like the genre. People who like EDM or metal, in turn, share basic sociodemographic traits, but differ with regard to personality traits. They are overall younger, more often men than women, and more often adopt an attitude of reorientation. EDM listeners, however, are more extraverted than people who do not like the genre, whereas metal fans are more open than fans of other music, but less conscientious and agreeable.

#### 3.2.2. Across sub-genre taste classes

Differences within genres are often larger than differences across genres, although the type and degree of effects vary depending on the specific genre (see Table 3). The greatest number of class differences, and the largest ones, exist in the classical and rock music groups; while metal and pop music classes show only some differences, and EDM classes are all relatively similar to each other. Multinomial regression models have shown that class differences within genres are related to between one and four of the ten variables (see Table 5). In this case, all models reached a moderate-to-substantial degree of explanatory power (Nagelkerke’s *R*^2^ from 0.144 to 0.270). Again, age, milieu-related attitude, and openness were the most relevant predictors, whereas class membership was not related to gender, neuroticism, or agreeableness.

The low, medium and mainstream preference classes differ often and consistently from the high preference class that was taken as reference. They are older (classical, pop, and rock), have lower openness scores (classical, pop, and rock), and more frequently hold a traditional attitude (EDM, metal, and rock). The sophisticated/hard classes in classical and rock are younger and less extravert than the high preference class (significant predictor in the classical model, with a similar tendency found in rock).

The other predictors are more genre-specific: education distinguishes between classes only in the classical and pop music groups, and in contrasting ways (all other classes are less well educated than the high preference classical class, whereas the youngest class in pop is better educated than the reference class); socioeconomic status and conscientiousness are only relevant for metal.

## 4. Discussion

In this study, we demonstrated that people who like the same musical genre do not necessarily share the same taste. Instead, separate taste classes emerge if attitudes toward sub-genres and -styles of a given genre are considered. Within groups of people reporting to like (i.e., ratings >3 on a five-point Likert scale) one of five different music genres—European classical music, electronic dance music (EDM), metal, pop, and rock—we found four to six such classes. Class patterns showed several similarities across genres, but also genre-specific differences. In general, the shapes of the profiles suggested two main types of classes: the first type consisted of three classes, the profiles of which were very similar to the group mean, but replicated it on a low, medium, or high level. A cross-check confirmed that this was not due to general response styles. The second type consisted of profile shapes that looked very different from the group mean. Here, we observed one to three differentiated classes that either liked (only) the more mainstream, easier, and softer sub-styles or disliked those and preferred sub-styles that were intellectually or perceptually more challenging. The majority of people who liked a genre belonged to the medium and high taste profiles, whereas the low and the differentiated classes were often (much) smaller. Only in the high preference class and the differentiated class with a preference for challenging and harder substyles did at least half the members assign a rating of 5 instead of only 4 to a given genre *χ*^2^-tests confirmed that there were significant differences between all classes for the distribution of 4 and 5 ratings (all *p* < 0.001) and did the high preference and hard/sophisticated classes differ from the others (see Supplementary Table S3). In that sense, these classes can be understood as representing the real “fans” of a genre, with people belonging to any of the other classes being interested only in parts of it.

The types of taste classes we found also correlated with differences in sociodemographic composition and personality traits in a consistent, i.e., genre-independent way. Such differences were often larger than differences across genre groups.

Given that our study included vastly different musical genres (popular and serious, mainstream and niche, those that attract age-homogenous audiences and others with age-heterogeneous ones) together with a representative participant sample, we assume that these types of taste classes reflect general tendencies that can also be found for other genres encompassing a variety of sub-styles. Specifically, the recognition of a mainstream/soft to challenging/hard dimension of taste differences may prove to be of general value for a genre-independent description of tastes.

In the following, we will first discuss the results of our study that can be elucidated by existing theories. Then, we will discuss those findings that differ from or expand existing knowledge.

### 4.1. Results in the light of existing theories

Across genres, both the sub-styles that represent the standard or core of the genre and those that can be seen as perceptually and cognitively less challenging were most liked. This overall liking pattern reflects two fundamental theories in empirical aesthetics, namely the preference for prototypicality (Martindale and Moore, 1988; North and Hargreaves, 2008, p. 84ss.) and the role of processing fluency for liking (Reber et al., 2004). These theories can also help to explain the emergence of classes that liked only the mainstream (i.e., prototypical), softer, and easier-to-process (i.e., more fluent) variants of a genre, particularly in classical music, EDM, metal, and rock. The relevant preference patterns, however, were associated with sociodemographic and personality traits, most importantly higher age, lower education, lower openness scores, and a tendency towards traditionalism. Therefore, the preference for prototypical and easy-to-process types of music is not solely a direct consequence of perceptual properties, but is also influenced by individual traits.

Those classes, however, that preferred perceptually and cognitively more challenging sub-styles and disliked the more mainstream ones seem to contradict theories of prototypicality and processing fluency. Here, arousal-based explanations (Berlyne, 1974; North and Hargreaves, 2008, p. 76ss.) or findings on the relationship between personality traits and preferences might be relevant (Rentfrow and McDonald, 2010). In our dataset, some evidence pointed towards underlying personality differences: in both classical music and rock, the sophisticated/hard classes had particularly high openness scores combined with the lowest extraversion scores of their group. Another potentially relevant trait that was not measured in our study but is known to be correlated with openness may be the need for cognition, which has already been discussed in the context of liking metal (Butkovic and Dopudj, 2017; Schmaltz et al., 2020). The finding of two contrasting types of musical tastes – one with a clear preference for easier forms of music, the other with a preference for more challenging ones – also reminds of the pleasure-interest model of aesthetic liking by Graf and Landwehr (2015, 2017). This model claims that pleasure and interest should be seen as two processes that can lead to aesthetic liking and are dependent on stimuli affordances and personality. Whereas Graf & Landwehr derived their theory from ratings of concrete stimuli, our results could be interpreted as showing the relevance of these two dimensions also on the meta-level of long-term attitudes towards music measured via (sub-)genre terms.

However, classes that disliked prototypical and easier sub-styles, but liked harder and more sophisticated variants were found for only some of the styles and consistently formed the smallest sub-group; this taste profile thus appears to be a relatively rare case. It nevertheless reflects discourses within some taste communities, e.g., that of EDM, where real fans or afficionados are expected to distinguish themselves by preferring challenging, avantgarde, and hip variants over mainstream types, and underground forms are developed to counter the popular variants (Jóri, 2021).

For pop music, a somewhat different picture emerged. This was in part related to the presented sub-styles having a different structure, and in part to the category of pop music in general, which can be defined by stylistic features much less clearly (Frith, 2001; Warwick, 2013). Our sub-genre categorization depended mainly on decades, which is a very common feature in pop music practices and discourses (e.g., there are many radio stations dedicated to pop music from only one or two decades). However, this approach did not allow for patterns of liking or disliking prototypicality to emerge. Rather, it afforded a response pattern that was more in line with theories about cohort effects of music preferences, most importantly the theory of song-specific age in popular music (Holbrook and Schindler, 1989; Kopiez et al., 2021) and of a reminiscence bump effect (Janssen et al., 2007).

That musical tastes might be grouped into types such as sophisticated/complex, mainstream/conventional, or hard/intense has already been proposed in other studies. One research strand is connected to the German sociologist Gerhard Schulze, who described three types of everyday aesthetics: the high culture scheme, the trivial scheme, and the tension scheme (Schulze, 1992–2000). Another direction is connected to music-psychological research on genre-independent structures of musical tastes (see, e.g., Rentfrow and Gosling, 2003; George et al., 2007). However, while these researchers’ interpretation was mostly based on genres or factors that comprised a number of musical (sub-)genres (see the MUSIC model of music taste proposed by Rentfrow et al., 2011; but see also Brisson and Bianchi, 2020, who demonstrate that already slight changes in the underlying data result in genres being grouped into different factors, thus challenging their interpretation), we show that similar types of taste exist already within one genre community (but see also Rentfrow et al., 2012, who found factors similar to the MUSIC model within jazz and rock). This suggests that genres (let alone groups of genres) are not the best level at which to account for structural differences between tastes because, amongst others, most genres cover a broad range of stylistic, formal, and expressive features that vary in how demanding and complex they are and have meaningful sub-branches that not all fans like equally well. Instead, one should consider those genre components that people actually like (or dislike) in order to describe and explain taste in a more nuanced way.

### 4.2. Results that expand or challenge existing knowledge

In light of the existing literature on musical taste, two of our results are particularly noteworthy. These are, first, the unexpected existence of a class that dislikes (almost) all sub-styles of a genre they reported to like. The existence of such, albeit small, low preference classes is in our view yet another indicator of the relatively poor meaning of a solely genre-related measurement of musical tastes. Also, the fact that the low preference classes are also characterized by lower openness scores indicates that it is not just a lower interest in that particular style, but in aesthetic artefacts in general (McCrae, 1993).

Another contribution of this study is its comparison of group differences in terms of sociodemographic composition and personality traits between and within genres and the finding that differences within genres were often larger than those between them. To date, research on musical taste and associated sociodemographic and personality differences has mostly focused on differences on the genre and genre-factor level, assuming that taste differences can be at least partly explained by these other differences.

The fact that in our study, differences between genre groups were often smaller than earlier studies lead one to expect is partly a consequence of allowing people to report their liking for more than one genre (as opposed to, e.g., North and Hargreaves, 2007a,b,c). This way, they fall into more than one genre group which leads to a relative similar composition of these groups.

Only when we compared people who liked a genre and those who disliked it did clearer differences appear. However, sociodemographic and personality traits predicted only preferences for classical music, EDM, and metal, and almost not at all pop and rock preferences. Dunn et al. (2012) also report small and inconsistent correlations between musical tastes, preferences, and the Big Five personality dimensions and conclude that both personality traits and musical tastes need to be captured more accurately.

Within genres, however, while pop and rock taste classes showed large differences from each other, the EDM and metal classes were very similar with regard to their sociodemographic composition and personality traits. We interpret this as reflecting a meaningful distinction between niche and mainstream preferences: while pop and rock listeners—being the largest groups—were quite representative of the general population, people who liked less popular genres were more distinct from it. Further, while fans of age-related niche genres in popular music (EDM, metal) were different from the general population, they were homogenous amongst themselves, whereas fans of mainstream genres in popular music (pop and rock) differed amongst themselves. Classical music, not being a popular music genre, is different in this regard, thus there are relatively large differences on both levels.

Sociodemographic or personality traits that predict belonging to a genre group or within-genre taste class were most often: age, milieu-related attitude, and openness; in some cases also: gender, education, extraversion, and agreeableness; and rarely to never: socioeconomic status, neuroticism, and conscientiousness. Except for the two Sinus-Milieu dimensions, which have never before been used in studies on musical tastes, the other factors are known to be of relevance and to distinguish between tastes on the levels of genre and genre factor (North and Hargreaves, 2008; Rentfrow and McDonald, 2010; Fricke and Herzberg, 2017). We extend these findings to differences within genre groups. While age, education, milieu-related attitude, and openness are similarly important on the genre and class levels, gender and agreeableness were only found to predict liking a genre, but not belonging to a within-genre taste class.

Two of these predictors may be discussed somewhat more exhaustively here, i.e., age and Sinus-Milieus. Age has been found to be a particularly important factor in previous research. Studies that used the MUSIC model of musical preferences have argued that different age groups prefer different types of music, and claimed an increase in liking so-called unpretentious and sophisticated music with age, but a decrease in liking so-called intense and contemporary music (see, e.g., Bonneville-Roussy et al., 2013), and have interpreted this as an effect of age, not of cohort. In our study, we found a comparable age-relation on the class level for those genres that had a large age range (classical, rock). In both cases, the oldest class preferred the soft/mainstream variants of the given genre, while the youngest group liked the hard/sophisticated sub-genres the most. Also, pop classes showed clear cohort effects. All classes most preferred pop music from the decade when they were around 20 years old. Taken together, this points to a combination of an age effect and a cohort effect in musical taste. The overall liking for a genre is to a large degree determined by musical socialization in adolescence and can be expected to remain relatively stable across the lifespan; whereas with age, preference within a given genre can change to adapt to individual changes in music-related needs and functions. Age dependencies of functions of music listening have already been shown (Schäfer and Sedlmeier, 2009; Hird and North, 2020).

In contrast to age, lifestyles have only rarely been associated with musical taste. For the German context, a classic study is Schulze’s *Erlebnisgesellschaft* (Schulze, 1992–2000), which described five milieus that differed in terms of their age, education, and which of three schemes of everyday aesthetics they preferred. The relationship between these milieus, schemes, and musical tastes has been examined further, on the basis of a representative German sample from 1997, by Otte (2008), who found differentiated evidence for the existence of the three schemes depending on age cohort, gender, and education level. The music-related item list that was used for their data collection also allowed some within-genre differentiation; e.g., for classical music, distinctions were made between pieces composed before and after 1900, opera, operetta, and musicals. Another large-scale attempt at associating lifestyles with musical tastes was a series of studies by North and Hargreaves (2007a,b,c) with a UK sample. Here, rather than sociologically defined milieus, individual lifestyle facets such as relationships, living arrangements, political beliefs, leisure and travel habits, and health were associated with being a fan of a certain music genre. Results were mapped onto sociological dimensions such as class schemata (upper-, middle-, lower-class; North and Hargreaves, 2007a or liberal—conservative; North and Hargreaves, 2007c). The use of Sinus-Milieus allows the analysis to go beyond these earlier results because they represent sociologically well-defined and -described lifestyle milieus that differ in their socioeconomic status and attitudes. We found that most taste groups and classes were distributed across all ten Sinus-Milieus, although in different ways, and that the attitude dimension, to a far higher degree than socioeconomic status, was the actual factor driving taste differences.

Overall, our findings provoke a theoretical question: What mechanism drives the relationships between musical taste and person-related factors? The most relevant predictors in our study were not fully independent from each other. Most importantly, age was negatively correlated with education and milieu-related attitude, which, in turn, were positively correlated with openness. In cases like the age-homogenous groups of EDM and metal listeners, however, attitude and, marginally, openness were still relevant for telling classes apart. This speaks to an underlying main factor of openness that could be inherited and/or acquired either by education or during the lifespan and that partly influences which genre(s) a person likes, but even more so what parts of it a person likes and how strongly. This contradicts somewhat other studies (e.g., Bonneville-Roussy et al., 2017) in which age differences are interpreted as direct age trends.

Age effects in the sense of cohort or generation effects (Smith, 1994; Glevarec et al., 2020) seem nevertheless to be meaningful in themselves, given that music, like all other cultural practices, is constantly changing and is an inherently historical phenomenon. Gender, socioeconomic status, and other personality traits, however, exert a much smaller influence and on only very few specific tastes.

### 4.3. Limitations and outlook

In light of the apparent importance of attitudes and personality traits, one limitation of this study is that it used a short, ten-item inventory to measure the Big Five personality traits (Rammstedt and John, 2007). This was necessary in the context of the already long questionnaire we were using. Although this and other very short versions of personality inventories have been shown to be reliable and have also been used in other studies on musical tastes (e.g., Rentfrow and Gosling, 2003; Greb et al., 2018), the long versions of the most important traits of openness, conscientiousness, and extraversion could potentially provide an even better idea of their relationship with certain musical tastes, as Dunn et al. (2012) have pointed out. In particular, only two of the six openness facets are covered by the BFI-10, including openness to aesthetics/artistic interest. Future studies are needed to examine whether similarly strong associations exist for other openness facets or for the overall openness measure.

Further, although our sample was representative, it consisted only of people living in Germany. Still, we expect our findings to hold also for other countries where the studied genres are widespread and form a similar constellation. We should be cautious, however, in assuming generalizability to countries with very different musical cultures without further corroborating empirical evidence. In particular, we expect the genre-specific findings to be dependent on the history and role of a genre within its respective musical genre world. The more abstract findings, however, such as the identification of within-genre taste classes, which differ with regard to the degree of liking sub-styles and the liking or disliking of mainstream/soft and sophisticated/hard variants, may be more likely to generalize across music cultures; and the same may be said for the dependency of the degree of differences in sociodemographic composition and personality traits from the popularity of a genre.

Overall, we think that our findings make a strong case for measuring (not only) musical tastes in a more nuanced way, accounting *inter alia* for sub-styles or other finer-grained differences within overall categories (a related example from the field of movie preference is Nave et al., 2020). While general categories such as genres are helpful for discussing music and taste in everyday life, they do not necessarily capture the musical and sociocultural differences of music very well (Eggert, 2022). Here, one should also consider those differentiations that are discussed by practitioners of the relevant types of music as well as of music theorists and musicologists. Eventually, better inventories for musical tastes are needed. This would allow us to better describe and, finally, to understand (music) taste as a cultural and socio-psychological phenomenon.

## Data availability statement

The raw data supporting the conclusions of this article will be made available by the authors, without undue reservation.

## Ethics statement

The studies involving human participants were reviewed and approved by Ethics council of the Max Planck Society. The patients/participants provided their written informed consent to participate in this study.

## Author contributions

MW-F designed the questionnaire, supervised the data collection, and formulated the overall research question. AS performed the latent profile analyses. AS and MW-F performed the other analyses and wrote the manuscript. All authors contributed to the article and approved the submitted version.

## Conflict of interest

The authors declare that the research was conducted in the absence of any commercial or financial relationships that could be construed as a potential conflict of interest.

## Publisher’s note

All claims expressed in this article are solely those of the authors and do not necessarily represent those of their affiliated organizations, or those of the publisher, the editors and the reviewers. Any product that may be evaluated in this article, or claim that may be made by its manufacturer, is not guaranteed or endorsed by the publisher.
