# Supplementary material for: You don’t know a person(’s taste) when you only know which genre they like: taste differences within five popular music genres based on sub-genres and sub-styles
Source: Front Psychol. 2023 Jun 7;14:1062146. doi: 10.3389/fpsyg.2023.1062146 (PMC10282133; doi:10.3389/fpsyg.2023.1062146)
Supplement: Supplementary file 1 [file Data_Sheet_1.pdf]

Supplementary Material

You don’t know a person(’s taste) when you only know which genre they like: taste differences within five popular music genres based on sub-genres and sub-styles

Anne Siebrasse* and Melanie Wald-Fuhrmann

* Correspondence: Anne Siebrasse: anne.siebrasse@ae.mpg.de

Supplementary Figure 1.

Distribution of Sinus Milieus Within Total Sample

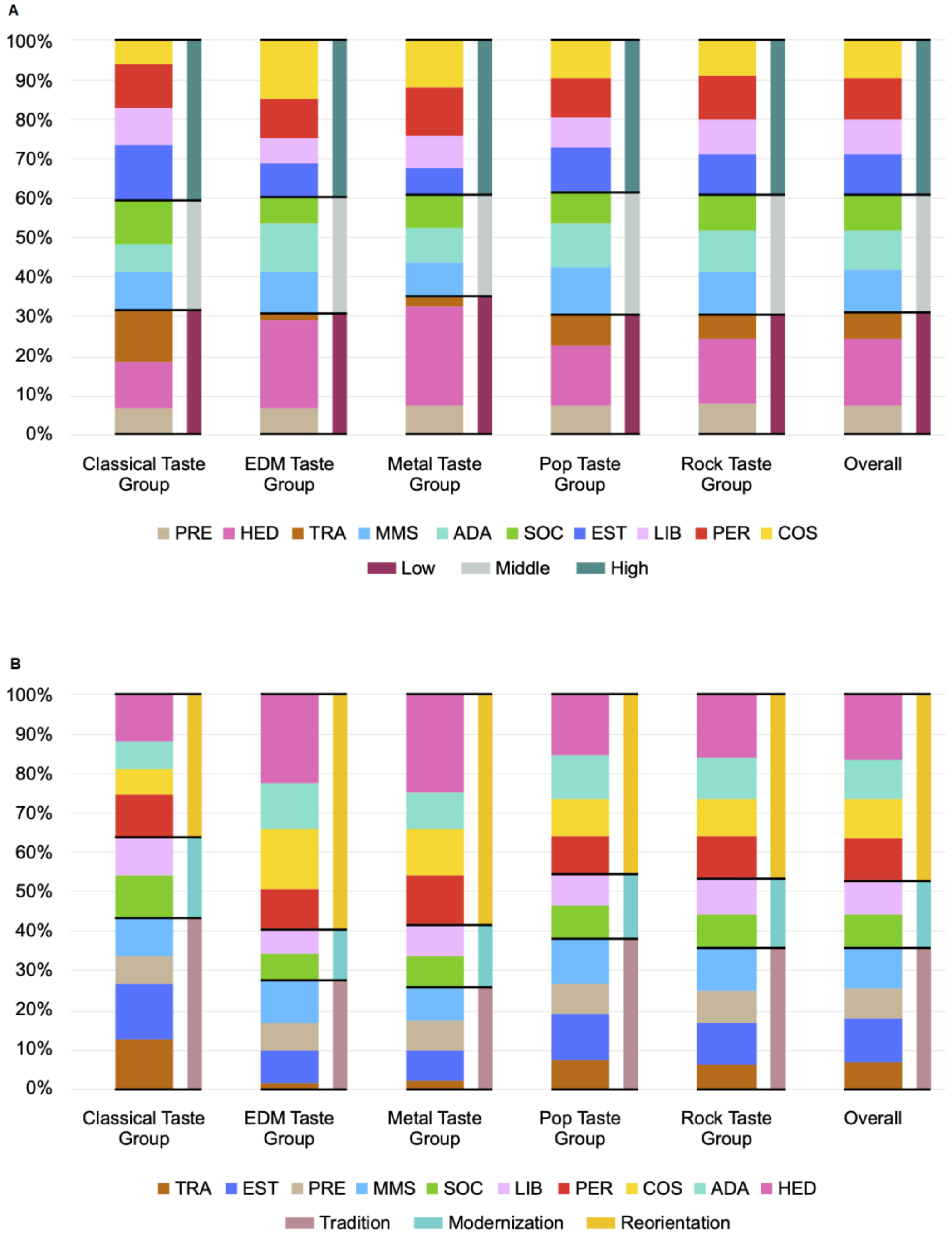

Note. A: Distribution of Sinus Milieus grouped along the attitude dimension. B: Sinus Milieus grouped along the dimension of socioeconomic status. PRE = Precarious, HED = Hedonists, TRA = Traditionals, MMS = Modern Mainstreamers, ADA = Adaptive Navigators, SOC = Social Ecologicals, EST = Established, LIB = Liberal Intellectuals, PER = Performers, COS = Cosmopolitan Avant-gardes.

Supplementary Figure 2.

Distribution of Sinus Milieus Within Classical Group

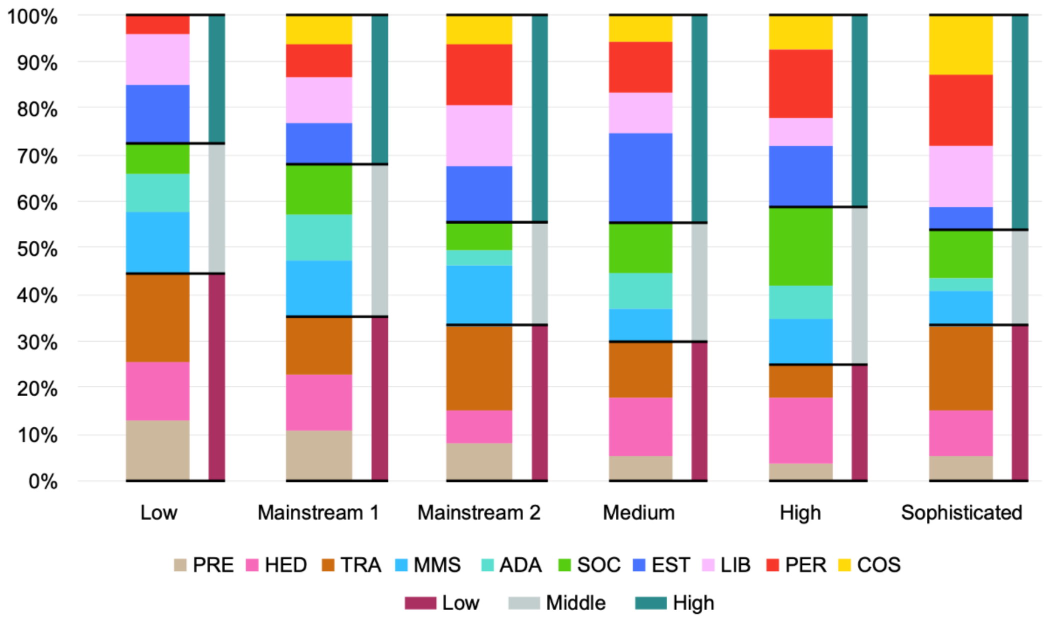

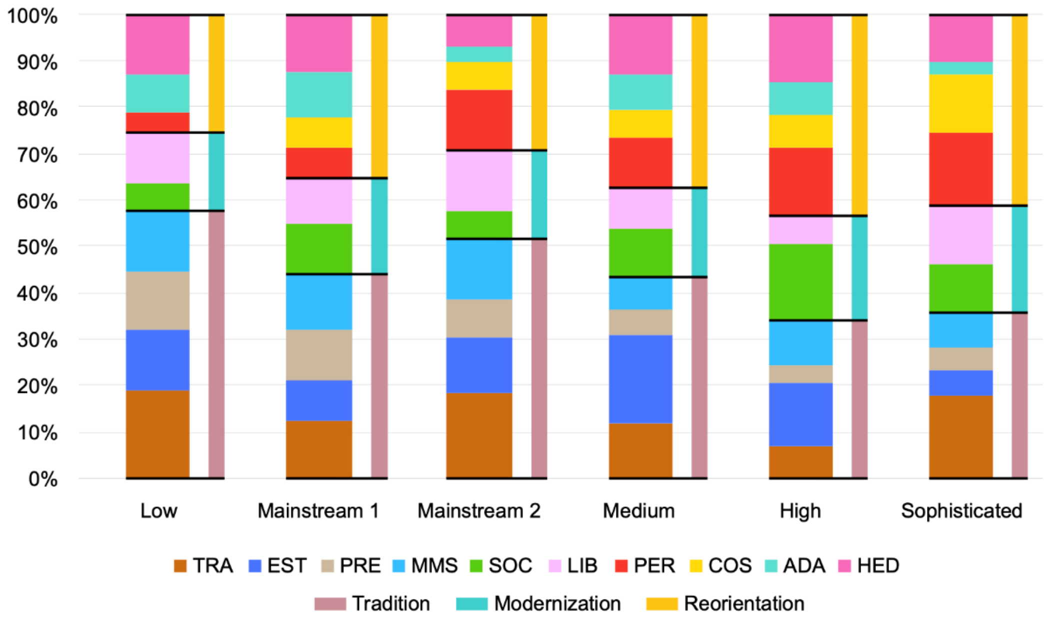

Note. A: Distribution of Sinus Milieus grouped along the attitude dimension. B: Sinus Milieus grouped along the dimension of socioeconomic status. PRE = Precarious, HED = Hedonists, TRA = Traditionals, MMS = Modern Mainstreamers, ADA = Adaptive Navigators, SOC = Social Ecologicals, EST = Established, LIB = Liberal Intellectuals, PER = Performers, COS = Cosmopolitan Avant-gardes.

Supplementary Figure 3.

Distribution of Sinus Milieus Within EDM Group

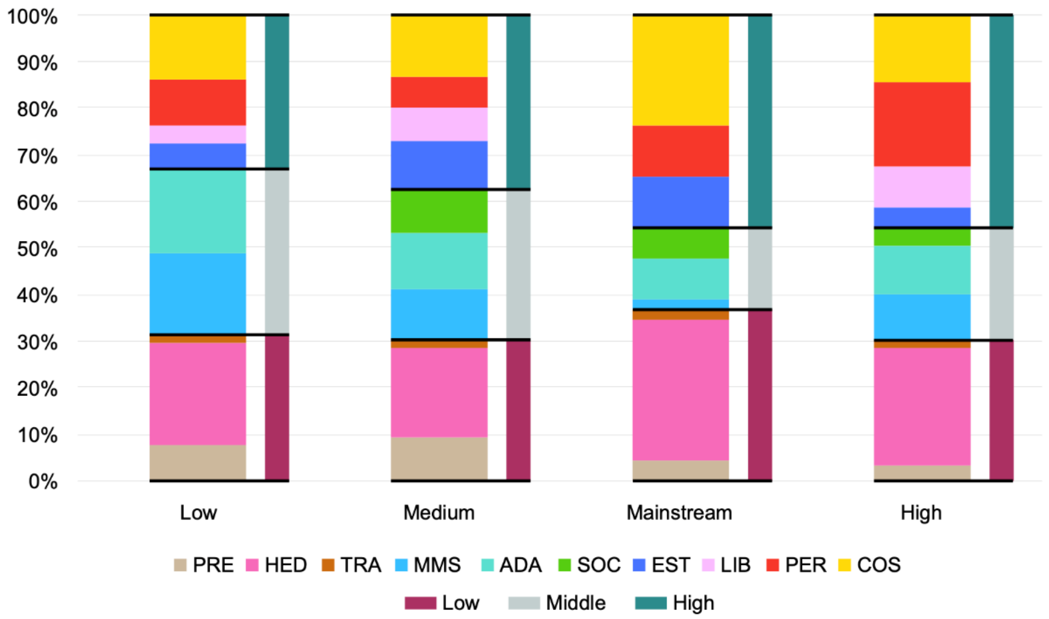

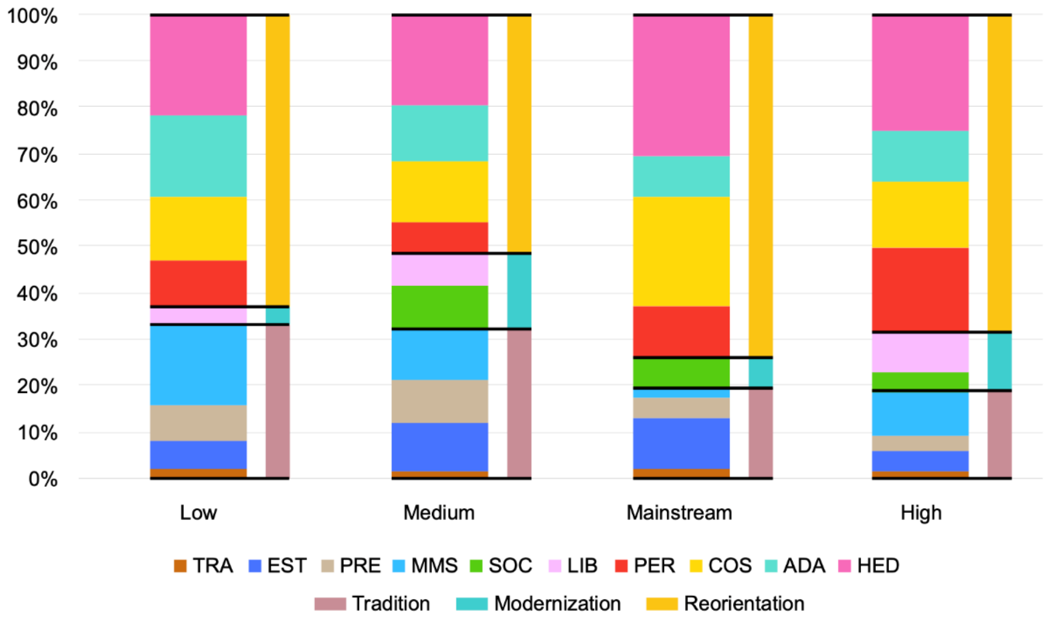

Note. A: Distribution of Sinus Milieus grouped along the attitude dimension. B: Sinus Milieus grouped along the dimension of socioeconomic status. PRE = Precarious, HED = Hedonists, TRA = Traditionals, MMS = Modern Mainstreamers, ADA = Adaptive Navigators, SOC = Social Ecologicals, EST = Established, LIB = Liberal Intellectuals, PER = Performers, COS = Cosmopolitan Avant-gardes.

Supplementary Figure 4.

Distribution of Sinus Milieus Within Metal Group

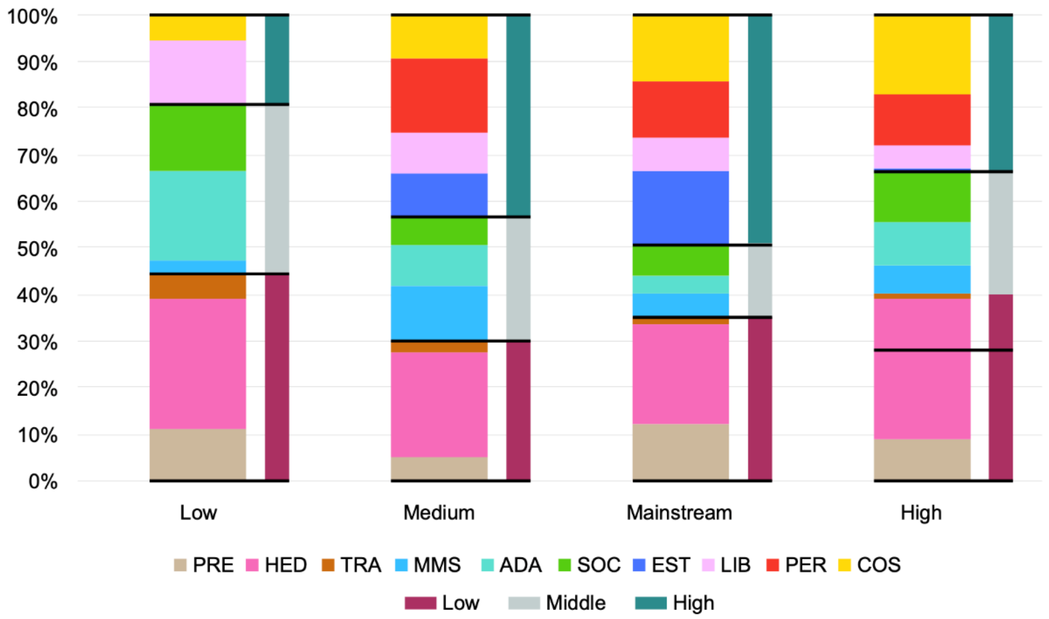

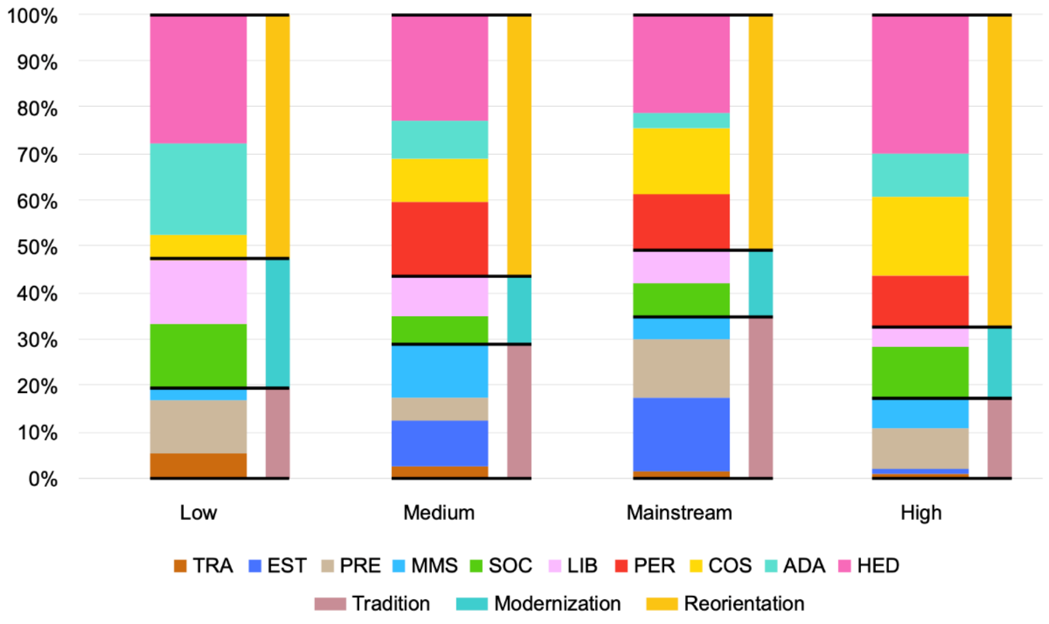

Note. A: Distribution of Sinus Milieus grouped along the attitude dimension. B: Sinus Milieus grouped along the dimension of socioeconomic status. PRE = Precarious, HED = Hedonists, TRA = Traditionals, MMS = Modern Mainstreamers, ADA = Adaptive Navigators, SOC = Social Ecologicals, EST = Established, LIB = Liberal Intellectuals, PER = Performers, COS = Cosmopolitan Avant-gardes.

Supplementary Figure 5.

Distribution of Sinus Milieus Within Pop Group

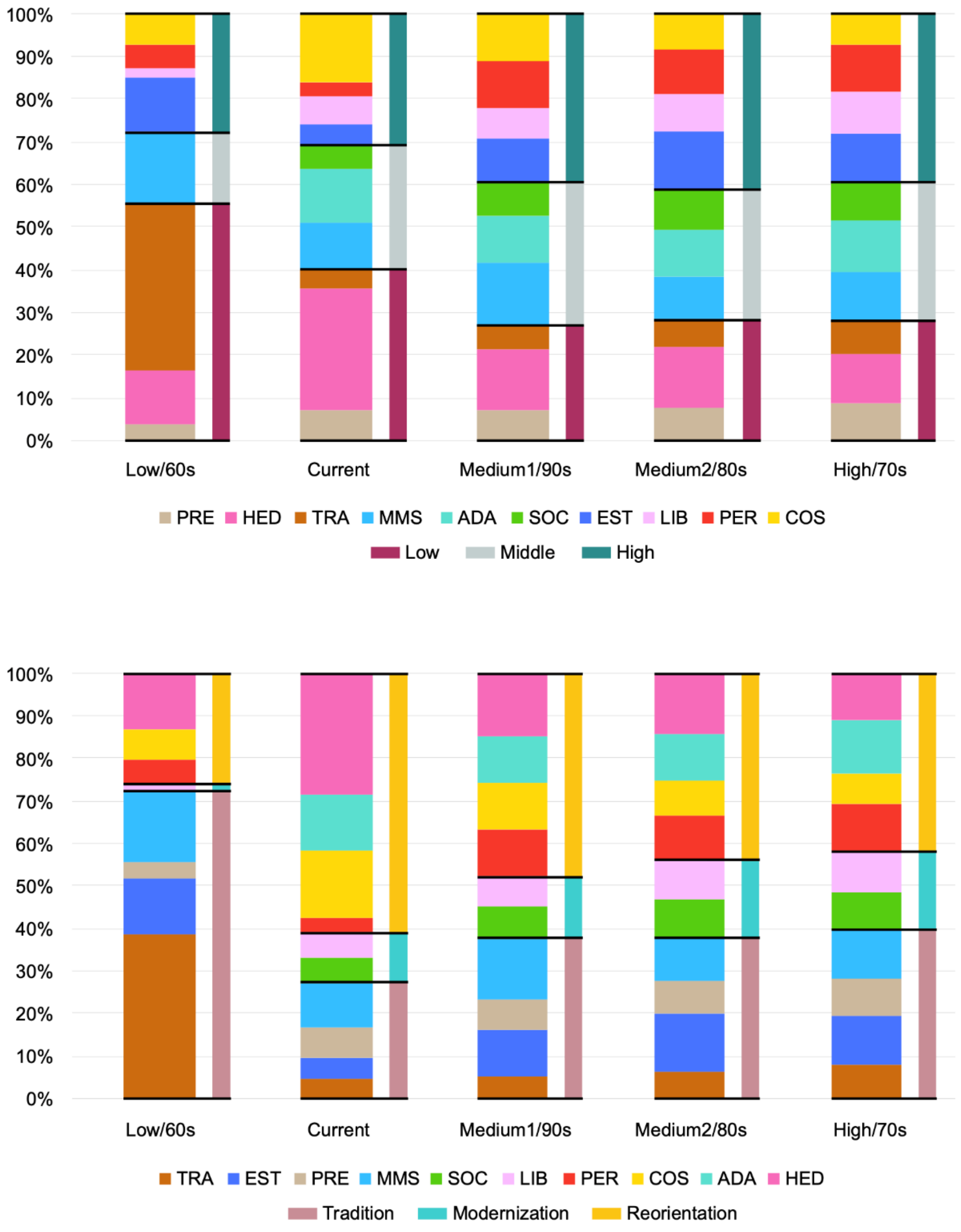

Note. A: Distribution of Sinus Milieus grouped along the attitude dimension. B: Sinus Milieus grouped along the dimension of socioeconomic status. PRE = Precarious, HED = Hedonists, TRA = Traditionals, MMS = Modern Mainstreamers, ADA = Adaptive Navigators, SOC = Social Ecologicals, EST = Established, LIB = Liberal Intellectuals, PER = Performers, COS = Cosmopolitan Avant-gardes.

Supplementary Figure 6.

Distribution of Sinus Milieus Within Rock Group

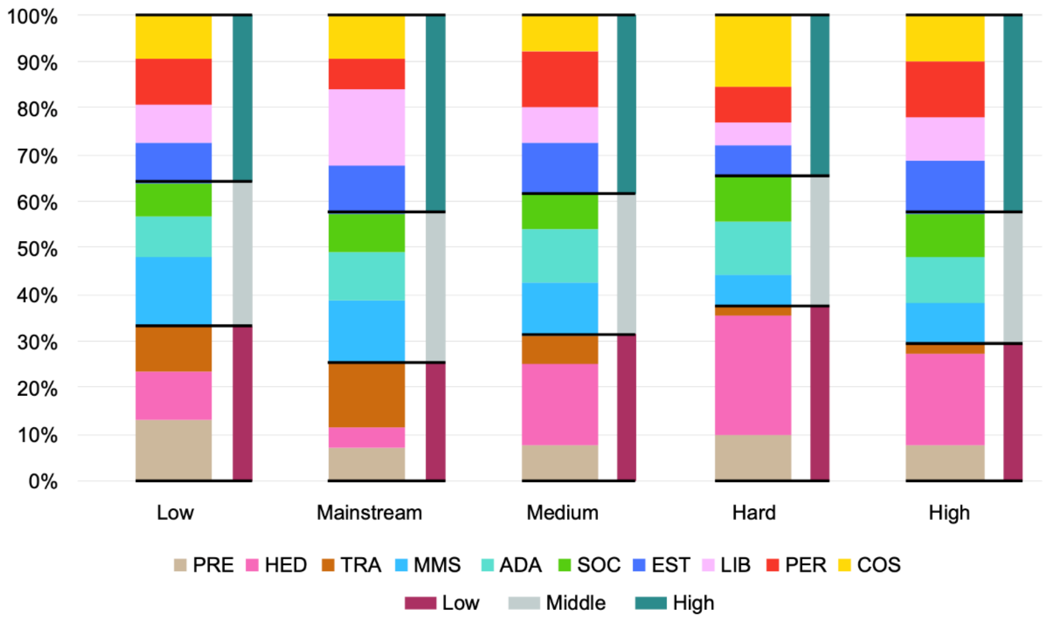

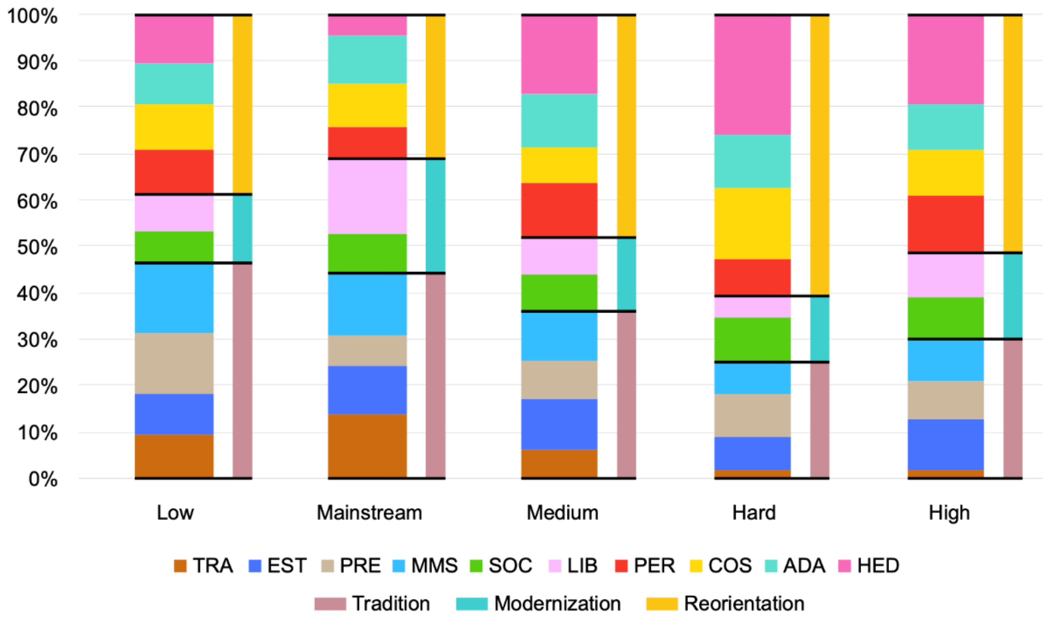

Note. A: Distribution of Sinus Milieus grouped along the attitude dimension. B: Sinus Milieus grouped along the dimension of socioeconomic status. PRE = Precarious, HED = Hedonists, TRA = Traditionals, MMS = Modern Mainstreamers, ADA = Adaptive Navigators, SOC = Social Ecologicals, EST = Established, LIB = Liberal Intellectuals, PER = Performers, COS = Cosmopolitan Avant-gardes.

Supplementary Figure 7.

Combination of Genres Liked by Participants

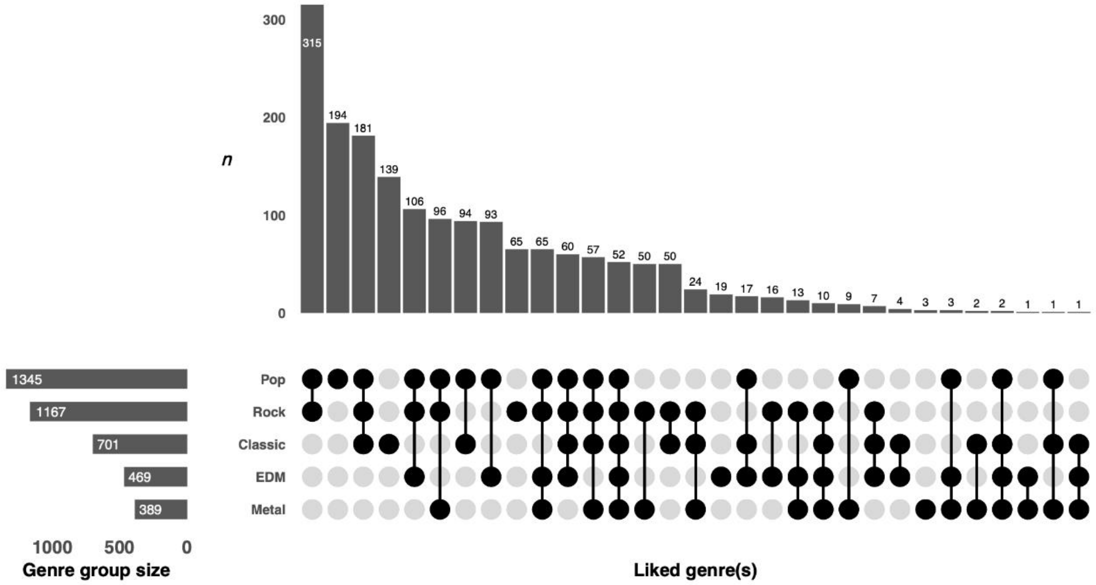

Supplementary Figure 8.

Class Membership Consistency for Participants Belonging to More than One Genre Group

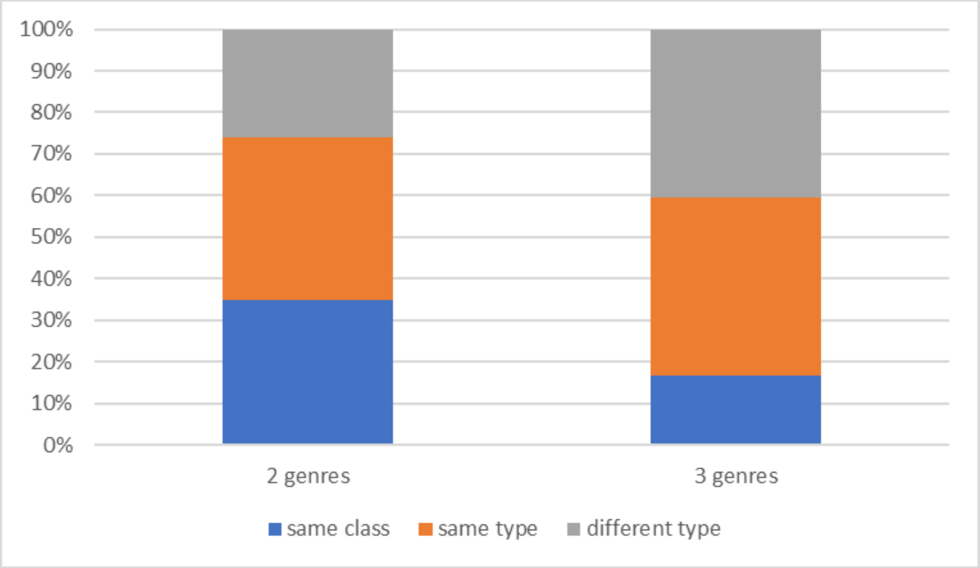

Supplementary Table 1

Liking Ratings and Percentages of Liking and Knowing on Genre Level

Genre	*n*	*M*	*SD*	% not knowing	% liking
Blues	1786	2.87	1.23	14.4	29.1
Country	1849	2.86	1.22	11.4	30.4
Electronic Dance Music	1637	2.63	1.40	21.5	24.5
Funk / Ska*	1287	2.38	1.18	38.3	11.5
Jazz	1819	2.43	1.22	12.8	18.2
Classical	1957	2.82	1.35	6.2	31.7
Metal	1615	2.38	1.41	22.6	19.5
Pop	2024	3.77	1.15	3.0	64.7
Rap / Hip Hop*	1740	2.49	1.36	16.6	22.4
Rock	1980	3.57	1.30	5.1	56.0
Schlager*	2050	3.16	1.43	1.7	47.4
Soul	1728	2.89	1.22	17.2	27.9
Non-European Music	1536	2.91	1.09	26.4	21.4
German Folk Music	2004	2.49	1.44	3.9	28.5

Note. Total N = 2,086. Genres without sub-styles are marked with *. Liking Ratings are based on five-point Likert scales.

Supplementary Table 2

Genre-Specific Attitudes and Behaviors for Genre Group and Taste Classes

	**Genre liking (*M*, *SD*)**	**Listening frequency (*M*, *SD*)**	**Substyle knowledge (*M*, *SD*)**
**Classical**			
total group	4.33 (0.47)	3.07 (0.96)	.84 (.26)
low	4.17 (0.38)	2.35 (0.81)	.79 (.26)
mainstream 1	4.14 (0.35)	2.69 (0.86)	.75 (.30)
mainstream 2	4.15 (0.36)	2.87 (0.84)	.77 (.28)
medium	4.27 (0.44)	3.00 (0.87)	.84 (.27)
high	4.69 (0.47)	3.72 (.90)	.95 (.13)
sophisticated	4.74 (0.44)	3.74 (0.75)	.94 (.10)
Δ	0.60	1.42	.20
**EDM**			
total group	4.41 (0.49)	3.44 (1.04)	.62 (.33)
low	4.35 (0.48)	3.10 (1.10)	.69 (.32)
medium	4.35 (0.48)	3.36 (1.01)	.57 (.34)
mainstream/soft	4.52 (0.51)	3.52 (1.07)	.64 (.25)
high	4.51 (0.50)	3.73 (0.98)	.69 (.33)
Δ	0.17	0.63	.12
**Metal**			
total group	4.45 (0.50)	3.54 (1.00)	.73 (.30)
low	4.17 (0.38)	2.75 (1.03)	.68 (.28)
medium	4.35 (0.48)	3.37 (0.93)	.72 (.33)
mainstream/soft	4.47 (0.50)	3.39 (0.98)	.70 (.28)
high	4.71 (0.46)	4.15 (0.77)	.79 (.25)
Δ	0.53	1.40	.11
**Pop**			
total group	4.46 (0.50)	4.01 (0.91)	.91 (.13)
low / 60s	4.07 (0.26)	3.69 (1.04)	.81 (.17)
current	4.47 (0.50)	4.11 (0.87)	.83 (.18)
medium / 90s	4.41 (0.49)	3.92 (0.89)	.88 (.15)
medium / 80s	4.46 (0.50)	3.95 (0.91)	.87 (.19)
high / 70s	4.53 (0.50)	4.15 (0.88)	.91 (.13)
Δ	0.46	0.46	.10
**Rock**			
total group	4.51 (0.50)	3.63 (1.00)	.77 (.25)
low	4.17 (0.37)	3.11 (1.03)	.72 (.27)
mainstream/soft	4.38 (0.49)	3.34 (0.89)	.71 (.22)
medium	4.45 (0.50)	3.49 (1.05)	.75 (.26)
sophisticated/hard	4.67 (0.47)	3.94 (0.89)	.86 (.18)
high	4.75 (0.43)	4.09 (0.79)	.83 (.23)
Δ	0.58	0.98	.15

Note. Genre preference and listening frequency are based on five-point Likert scales. Sub-genre knowledge is a mean value normalized to range from 0 to 1.

Supplementary Table 3

Distribution of Liking Ratings on Genre Level

Genre Group	Rating		Class						
			low	medium	high	mainstream 1	mainstream 2	sophisticated / hard	Total per genre group
Classical									
	4	% within class	83.3 $_{a}$	73.1 $_{a}$	31.4 $_{b}$	86.1 $_{a}$	84.8 $_{a}$	25.6 $_{b}$	66.8
	5	% within class	16.7 $_{a}$	26.9 $_{a}$	68.6 $_{b}$	13.9 $_{a}$	15.2 $_{a}$	74.4 $_{b}$	33.2
EDM									
	4	% within class	64.7 $_{a, b}$	65.5 $_{a}$	48.8 $_{b}$	47.8 $_{a, b}$			59.3
	5	% within class	35.3 $_{a, b}$	34.5 $_{a}$	51.2 $_{b}$	52.2 $_{a, b}$			40.7
Metal									
	4	% within class	83.3 $_{a}$	65.1 $_{a, b}$	29.1 $_{c}$	52.6 $_{b}$			54.8
	5	% within class	16.7 $_{a}$	34.9 $_{a, b}$	70.9 $_{c}$	47.4 $_{b}$			45.2
Pop			low/60s		high/70s	90s	80s	current	
	4	% within class	92.6 $_{a}$		47.2 $_{b}$	59.2 $_{c}$	53.8 $_{b, c}$	52.5 $_{b, c}$	54.5
	5	% within class	7.4 $_{a}$		52.8 $_{b}$	40.8 $_{c}$	46.2 $_{b, c}$	47.5 $_{b, c}$	45.5
Rock									
	4	% within class	83.3 $_{a}$	55.2 $_{b}$	24.6 $_{c}$	62.1 $_{b}$		32.7 $_{c}$	48.8
	5	% within class	16.7 $_{a}$	44.8 $_{b}$	75.4 $_{c}$	37.9 $_{b}$		67.3 $_{c}$	51.2

Note. Each subscript letter denotes a subset of the class membership categories (per genre) whose column proportions do not differ significantly from each other at the .05 level.
